# Metagenome-Based Functional Differentiation of Gut Microbiota and Ecological Adaptation Among Geographically Distinct Populations of Przewalski’s Gazelle (*Procapra przewalskii*)

**DOI:** 10.3390/microorganisms13112513

**Published:** 2025-10-31

**Authors:** Jingjie Zhang, Feng Jiang, Xiaohuan Li, Pengfei Song, Tongzuo Zhang

**Affiliations:** 1 State Key Laboratory of Plateau Ecology and Agriculture, Qinghai University, Xining 810016, China; jingjiezhang1@163.com (J.Z.);; 2 Qinghai Provincial Key Laboratory of Animal Ecological Genomics, Xining 810001, China; 3 Key Laboratory of Adaptation and Evolution of Plateau Biota, Northwest Institute of Plateau Biology, Chinese Academy of Sciences, Xining 810001, China

**Keywords:** Przewalski’s gazelle, metagenomics, gut microbiota, functional differentiation, ecological adaptation

## Abstract

Przewalski’s gazelle (*Procapra przewalskii*) is an endangered ungulate endemic to the Qinghai–Tibet Plateau, with a small population size and exposure to multiple ecological pressures. Its gut microbiota may play a crucial role in host environmental adaptation. To investigate the functional divergence of gut microbial communities, we performed high-throughput metagenomic sequencing on 105 wild fecal samples collected from 10 geographic regions around Qinghai Lake. The results revealed significant regional differentiation in key functional modules related to metabolism, antibiotic resistance mechanisms, and virulence-associated pathways. All populations showed enrichment in core metabolic pathways such as carbohydrate and amino acid metabolism, with carbohydrate-active enzymes dominated by glycoside hydrolases (GHs) and glycosyltransferases (GTs), exhibiting overall functional conservation. Although populations shared many antibiotic- and virulence-related reference genetic markers, the marker composition associated with distinct resistance mechanisms and pathogenic processes exhibited clear population-specific patterns, suggesting differential microbial responses to local environmental pressures. Correlation network analysis further identified core taxa (e.g., *Arthrobacter* and Oscillospiraceae/Bacteroidales lineages) as key genera linking community structure with core metabolic, resistance-related, and virulence-associated marker functions. Overall, the gut microbiota of Przewalski’s gazelle exhibits a complex spatially structured functional differentiation, reflecting host–microbiome co-adaptation under region-specific ecological pressures. These findings provide critical methodological and theoretical support for microecological health assessment and regionally informed conservation management of this endangered species.

## 1. Introduction

The Qinghai–Tibet Plateau harbors a unique and fragile alpine ecosystem, where extreme environmental conditions pose significant challenges to the survival and adaptation of living organisms. Przewalski’s gazelle (*Procapra przewalskii*), a flagship species of this plateau ecosystem and an endemic species to China, is listed as a Class I nationally protected animal. Historically, the species had a wide distribution, but it is now confined to small, fragmented populations around Qinghai Lake and its surrounding areas [1,2]. With a current total population of fewer than 4000 individuals, its restricted distribution and small population size render it highly susceptible to environmental changes and disease disturbances.

The gut microbiota plays a critical role in animal health and ecological adaptability. It assists the host in the breakdown of substances and nutrient metabolism [3], and also plays a key role in host-specific immune regulation [4]. Moreover, it is increasingly recognized as a crucial modulator of the host’s capacity to cope with environmental changes [5]. In recent years, studies on the gut microbiota of wild animals, particularly endangered species, have provided new insights into the physiological mechanisms underlying their adaptation to extreme environments, offering a theoretical foundation at the microecological level for conservation strategies. For instance, previous research has suggested that the gut microbiota of the giant panda (*Ailuropoda melanoleuca*), which primarily consumes highly fibrous bamboo, may play a vital role in the digestion of otherwise indigestible plant components and in enhancing protein utilization under limited protein availability [6]. Similarly, species such as the Yunnan snub-nosed monkey (*Rhinopithecus bieti*) and reindeer (*Rangifer tarandus valentinae*), which inhabit cold environments, have been found to rely on the functional capabilities of their gut microbiota to adapt to low-temperature conditions [7].

Currently, populations of Przewalski’s gazelle are distributed across several regions characterized by pronounced ecological heterogeneity, with notable differences in vegetation types [8] and the intensity of human disturbance. These environmental differences may drive divergence in the composition and functional profiles of their gut microbiota. Previous studies have shown that Przewalski’s gazelles exhibit distinct patterns of microbial diversity across different distribution areas [8]. Moreover, comparative analysis with the more widely distributed Tibetan gazelle revealed that inter-population variation in gut microbiota diversity is significantly greater in Przewalski’s gazelle, potentially reflecting limited gene flow and reduced contact among populations [9]. Therefore, this study aims to further investigate the functional variability of gut microbiota in Przewalski’s gazelle under different ecological contexts. By uncovering how host-microbiota co-adaptation influences ecological adaptation strategies, we seek to identify region-specific microbial functions and provide a microecological perspective to inform more targeted and effective conservation and management strategies.

Therefore, this study aims to conduct a comparative analysis of the functional composition of gut microbiota in Przewalski’s gazelle populations from different geographic regions using high throughput metagenomic sequencing. Specifically, the study seeks to address the following questions: (i) What are the core and variable functional traits of the gut microbiota among geographically distinct populations of Przewalski’s gazelle? (ii) Which microbial functional modules play key roles in the survival and ecological adaptation of this species? The findings will provide a scientific basis for health assessment and the development of region-specific conservation strategies for this endangered species.

## 2. Materials and Methods

### 2.1. Sample Collection and Processing

Based on the current distribution of Przewalski’s gazelle, fecal samples were collected from ten distinct geographic regions during April to May 2023 (Table S1). Compared with previous sampling efforts [8], this study expanded coverage by including two previously under-sampled areas, Talexuanguo (TLXG) and Yuanzhe (YZ), and excluded the semi-captive Jiangxigou Rescue Station (JXG), thereby providing a more comprehensive representation of wild populations for analyzing functional differences in gut microbiota across regions. A total of 105 fresh fecal samples were collected using sterile disposable polyethylene gloves and sterile sampling bags to prevent cross-contamination. Samples were immediately placed in liquid nitrogen for transport to the laboratory and subsequently stored at −80 °C for downstream high-throughput DNA sequencing analysis. To minimize the inherent risk of pseudo-replication in noninvasive fecal sampling, we implemented strict spatial separation in the field: a distance of ≥500 m between different subpopulations/subsampling subareas and a ≥50 m minimum distance between distinct fecal piles within the same subarea. We also applied morphological screening (including pellet size, shape, and color) to avoid collecting samples likely originating from the same defecation event. Given that fecal DNA cannot be unequivocally matched to specific individuals under field conditions, we report the number of samples rather than individuals, and treat each fecal sample as an independent observational unit in statistical analyses.

### 2.2. DNA Extraction, Amplification, and Sequencing

The total microbial DNA was extracted from the fecal samples using the MagBeads FastDNA Kit for Soil (116564384, MP Biomedicals, 6 Thomas, Irvine, CA, USA) according to the manufacturer’s instructions. After DNA extraction, the concentration and purity of the DNA were assessed, and DNA integrity was evaluated using 1% agarose gel electrophoresis. Genomic DNA libraries were constructed following the standard Illumina TruSeq DNA library preparation protocol (Illumina TruSeq DNA Sample Preparation Guide). DNA was fragmented using a Bioruptor, and libraries were prepared through end repair, adapter ligation, and PCR amplification. The resulting libraries were quantified using the Qubit 4 fluorometer. Multiplexed DNA libraries were normalized to a final concentration of 10 nM and pooled in equal volumes. The pooled libraries were then diluted to the appropriate concentration and subjected to high-throughput sequencing on the NovaSeq X Plus platform using a paired-end sequencing strategy (2 × 150 bp).

### 2.3. Metagenomic Quality Control and Assembly of Experimental Samples

The raw sequencing data in this study were subjected to quality control using (v0.23.2) [10], which involved the removal of adapter sequences and low-quality reads, as well as statistical evaluation of sequencing quality before and after filtering. Subsequently, high-quality reads from each sample were assembled into contigs using MEGAHIT (v1.1.2) [11], with only contigs ≥300 bp retained by default. Open reading frames (ORFs) were identified from the assembled contigs using Prodigal (v2.6.3) [12], and both nucleotide and corresponding amino acid sequences of predicted coding regions were extracted. We clustered all predicted gene sequences from all samples using CD-HIT [13] (v4.7) with parameters of 90% sequence identity and 90% coverage. For each cluster, the longest gene was selected as the representative to construct a nonredundant gene catalog. We then used SOAPaligner [14] (SOAP2 v2.21 release) to align the high-quality reads of each sample to the nonredundant gene catalog at 95% identity, and quantified gene abundances per sample accordingly.

### 2.4. Metagenomic Annotation and Analysis of Experimental Samples

After quality control and construction of the nonredundant gene catalog, we performed protein-level functional annotation. For KEGG (Release 103.1, July 2022), amino acid sequences were aligned to the KEGG database using Diamond [15] (v2.0.13) with the BLASTP algorithm (e-value ≤ 1 × 10^−5^) to obtain pathway and other functional category abundances. For CAZy (v8; accessed on 30 November 2023), sequences were aligned with HMMER (v3.1b2; e-value ≤ 1 × 10^−5^) to obtain carbohydrate-active enzyme (CAZyme) annotations, and CAZyme abundances were calculated by summing the abundances of genes assigned to each family.

For CARD (v3.0.9) and VFDB (https://www.mgc.ac.cn/VFs/, accessed on 3 July 2020), sequences were aligned with Diamond; ARG function abundances were computed as the sum over genes assigned to each function, and virulence factors were taken from VFDB hits under the same parameters. These procedures produced taxonomic abundance tables across multiple ranks and functional abundance tables for the respective categories, which were used for subsequent statistical analyses and visualization. 

### 2.5. Data Analysis and Visualization

We quantified and visualized functional abundances of regional Przewalski’s gazelle gut microbiomes using KEGG, CAZy, CARD, and VFDB. Venn diagrams summarized the numbers and proportions of region-unique and shared antibiotic resistance ontology (ARO) features. Heatmaps and bar charts depicted the functional composition of KEGG Level 2 pathways, CARD antibiotic classes, and VFDB virulence factors, while Circos plots integrated the class-level composition and relative proportions of CAZy across regions. Between-group separation was assessed with ANOSIM (Bray–Curtis; 999 permutations), reporting pairwise R and *p* values. For multi-group comparisons of functional profiles, we applied the Kruskal–Wallis test to each feature (KEGG Level-2 pathways; CAZy families) and quantified effect size with epsilon-squared, $\varepsilon^{2}=(H-k+1)/(n-k)$, where H is the KW statistic, k is the number of groups, and n is the total sample size. Uncertainty was characterized via within-group percentile bootstrap (600 resamples with replacement, fixed random seed) to obtain 95% confidence intervals (2.5–97.5%). *p* values were FDR-adjusted to q values, and we highlighted the top 10 features with both high abundance and pronounced between-group variation.

Differential features were further identified using LEfSe (LDA > 2) based on KEGG Level 2, CAZy class, CARD antibiotic classes, and VFDB virulence factors. Using sample-level taxon–function mappings, we quantified and visualized species contributions to functional abundances (KEGG Level 2; CAZy class), emphasizing dominant taxa. To examine links between ARG/virulence functions and community structure, the genus-level NR_Origin table was treated as the microbial factor (Factor 1) and CARD (antibiotic classes) plus VFDB (virulence factors) as functional factors (Factor 2), retaining the top 10 genera and top 30 functional categories by abundance. Spearman correlations were computed between taxa and functions (|*r*| ≥ 0.6; *p* < 0.05); significant associations defined network edges, with taxa and functions as nodes visualized by abundance and centrality. Finally, linear regressions between taxonomic and functional β-diversity metrics (KEGG Level-2; CAZy class) evaluated community-level coupling.

## 3. Results

### 3.1. Functional Annotation Analysis of Gut Microbiota in Different Groups of Przewalski’s Gazelle

**Figure 1:**
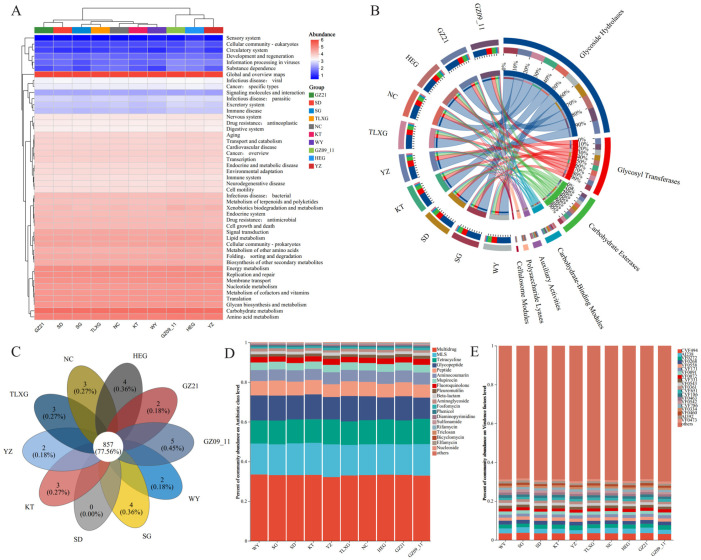
Functional composition of Przewalski’s gazelle gut microbiota across regions. (**A**) Functional composition at KEGG Level 2; (**B**) Functional composition of carbohydrate-active enzyme (CAZymes) classes based on the CAZy database; (**C**) Composition of antibiotic resistance ontology (ARO) functions annotated by the CARD database; (**D**) Composition of antibiotic resistance classes based on the CARD database; (**E**) Composition of virulence factor functions annotated by the VFDB database.

The functional heatmap based on KEGG Level 2 annotations (Figure 1A) revealed a broadly conserved functional core across regions, with highest relative abundances in carbohydrate, amino-acid, and energy metabolism. Within Genetic Information Processing, translation and DNA replication/repair were also prominent. Further analysis based on CAZy database annotations (Figure 1B) indicated carbohydrate-active enzymes were dominated by Glycoside Hydrolases (GHs) and Glycosyl Transferases (GTs), with GH accounting for ~53.30% of assignments, whereas CE, PL, AA, and CBM comprised smaller fractions. Overall, despite minor regional fluctuations, carbohydrate-metabolism–related functions were strongly consistent among regions.

Based on the CARD Antibiotic Resistance Ontology (ARO) annotation, the gut microbiota of Przewalski’s gazelle populations shared a large common resistome: 857 co-occurring AROs (77.56%) were detected across all regions, while region-specific AROs were present in most groups (the SD population being an exception) (Figure 1C). At the antibiotic-class level (Figure 1D), database-inferred ARG categories (CARD) were largely concentrated in a small set of classes—multidrug, macrolide–lincosamide–streptogramin (MLS), tetracycline, glycopeptide, and peptide—yielding broadly similar class-level profiles across regions. VFDB-based composition (Figure 1E) likewise showed a stable framework of homology-based virulence-factor categories, with LOS (CVF494), fibronectin-binding proteins (AI238), and the FbpABC transport system (VF0272) relatively abundant in multiple regions; these database-inferred markers do not imply expression or pathogenicity. Overall, these results support a pattern dominated by a shared core with only minor regional differences: metabolic functions and CAZyme classes are highly conserved, whereas ARG and virulence categories are broadly similar at the class level when interpreted as reference-gene homolog repertoires.

### 3.2. Analysis of Functional Beta Diversity of Gut Microbiota in Different Populations of Przewalski’s Gazelle

**Figure 2:**
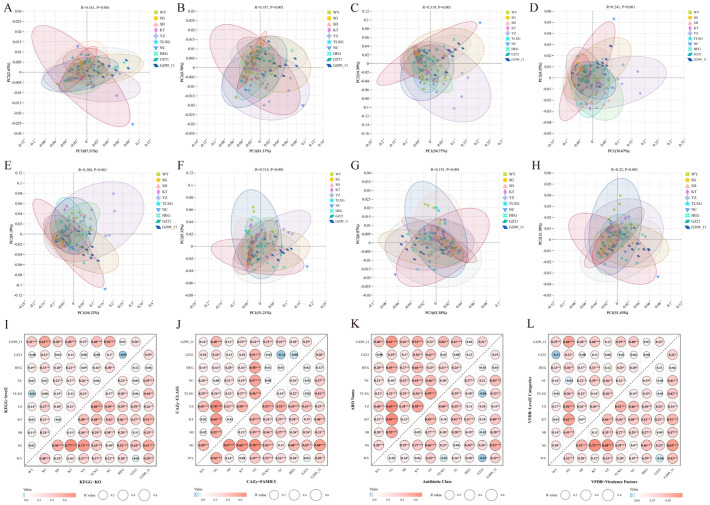
Differences in gut microbial functions of Przewalski’s gazelle across regions. (**A**,**B**) Functional differences at KEGG Level-2 and Level-3; (**C**,**D**) Group-level differences in carbohydrate-active enzymes (CAZy) at the class and family levels; (**E**,**F**) Group-level differences in antibiotic resistance ontology (ARO) and antibiotic class annotations based on CARD database; (**G**,**H**) Functional differences in virulence factors and Level 2 categories based on VFDB database; (**I**–**L**) Pairwise differential analyses across groups for KEGG, CAZy, CARD, and VFDB at two hierarchical levels, indicating the statistical significance of each group-to-group test. Significance: *p* < 0.05 (*), *p* < 0.01 (**), *p* < 0.001 (***).

Distinct regional differentiation was observed in the functional profiles of the gut microbiota of Przewalski’s gazelle across multiple databases and hierarchical levels. Regarding metabolic functions, both KEGG (Levels 2 and 3) and CAZy (class and family levels) revealed significant intergroup variation and clear clustering separation. In most pairwise permutation tests, the R values ranged from 0.2 to 0.7 and were statistically significant, indicating systematic differences in substrate utilization and metabolic potential among regions (Figure 2A–D,I,J). For reference databases associated with potential health-related functions, clear regional heterogeneity was also identified in CARD and VFDB annotations. In CARD, both the ARO and antibiotic-class levels exhibited region-specific distributions of antibiotic-related gene markers, whereas in VFDB, the virulence-factor and Level 2 annotations likewise displayed regionally differentiated profiles (Figure 2E–H,K,L). Based on metagenomic alignments to these two reference databases, the results indicate that the gut microbiomes harbor homology-based gene sets associated with antibiotic resistance and virulence, reflecting regional variation in genetic potential rather than expression or phenotype. Further pairwise comparisons showed that regions such as GZ09_11, HEG, YZ, and TLXG exhibited broad and significant differentiation from most other regions at both the metabolic (KEGG/CAZy) and candidate gene-marker (CARD/VFDB) levels (Figure 2I–L). Collectively, these patterns suggest that regional environmental conditions and host ecological contexts jointly shape both the metabolic potential and the composition of antibiotic- and virulence-associated gene pools within the gut microbiota of Przewalski’s gazelle. 

### 3.3. Differential Analysis of Gut Microbial Functions Among Przewalski’s Gazelle Populations

**Figure 3:**
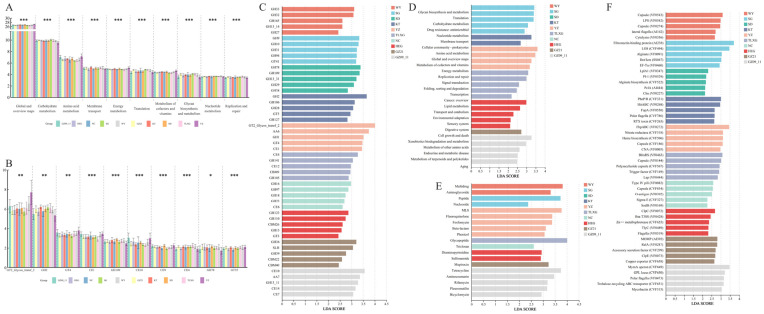
Functional differentiation of gut microbiota among geographic populations of Przewalski’s gazelle across databases. (**A**) Top 10 significantly different KEGG Level 2 pathways among groups; (**B**) Top 10 significantly different CAZy Family functions among groups; (**C**) Top 5 group-specific marker functions (LDA > 2) at the CAZy Family level identified by LEfSe; (**D**) Top 5 group-specific marker functions (LDA > 2) at the KEGG Level2 level identified by LEfSe; (**E**) Top 5 group-specific marker functions (LDA > 2) at the CARD Antibiotic class level identified by LEfSe; (**F**) Top 5 group-specific marker functions (LDA > 2) at the VFDB virulence-factor level identified by LEfSe. Significance: *q* < 0.05 (*), *q* < 0.01 (**), *q* < 0.001 (***), where q denotes Benjamini–Hochberg FDR–adjusted p-values computed across features within each functional level.

To further elucidate functional differences across geographically distinct populations of Przewalski’s gazelle, we compared the KEGG Level 2 and CAZy Family tiers (*k* = 10 groups; *df* = 9; *n* = 105). At KEGG Level 2, multiple metabolism-related pathways showed highly significant among-population variation with large effects. For example, Global and overview maps (*q* = 3.58 × 10−^8^; ε^2^ ≈ 0.54), Amino acid metabolism (*q* = 1.06 × 10^−7^; ε^2^ ≈ 0.59), Energy metabolism (*q* = 9.99 × 10^−7^; ε^2^ ≈ 0.58), Translation (*q* = 1.14 × 10^−6^; ε^2^ ≈ 0.55), and Metabolism of cofactors and vitamins (*q* = 1.23 × 10^−8^; ε^2^ ≈ 0.66) were all strongly differentiated among populations. Additional but somewhat smaller effects were observed for Glycan biosynthesis and metabolism (*q* = 3.36 × 10−^5^; ε^2^ ≈ 0.54), Membrane transport (*q* = 7.38 × 10^−5^; ε^2^ ≈ 0.38), Nucleotide metabolism (*q* = 7.38 × 10^−5^; ε^2^ ≈ 0.46), and Replication and repair (*q* = 4.13 × 10^−4^; ε^2^ ≈ 0.58) (Figure 3A, Table S2). These results indicate that among-population differences are concentrated in metabolic functions, with additional variation in non-metabolic categories such as transport and translation.

At the CAZy Family level, numerous high-abundance enzyme families of glycoside hydrolases (GHs), glycosyltransferases (GTs), and carbohydrate esterases (CEs) exhibited significant or highly significant differences. Representative families include GT2_Glycos_transf_2 (*q* = 2.59 × 10^−3^; ε^2^ ≈ 0.44), GH2 (*q* = 1.03 × 10^−3^; ε^2^ ≈ 0.50), GT4 (*q* = 6.26 × 10^−3^; ε^2^ ≈ 0.52), CE1 (*q* ≈ 7.13 × 10^−5^; ε^2^ ≈ 0.63), GH109 (*q* ≈ 8.6 × 10^−5^; ε^2^ ≈ 0.31), CE10 (*q* = 2.25 × 10^−5^; ε^2^ ≈ 0.37), CE4 (*q* = 8.06 × 10^−6^; ε^2^ ≈ 0.59), GH78 (*q* = 4.07 × 10^−2^; ε^2^ ≈ 0.40), and GT35 (*q* = 2.77 × 10^−4^; ε^2^ ≈ 0.61) (Figure 3B, Table S2). Collectively, these functional shifts highlight distinct ecological adaptations of gut microbiota among populations, particularly in carbohydrate metabolism and associated enzymatic activities, potentially reflecting long-term selection by differences in plant-derived substrates and fiber structures.

To identify the functional specificity of gut microbiota in different Przewalski’s gazelle populations, we conducted LEfSe analysis (LDA score > 2) to compare significantly enriched functional genes across groups (Figure 3C–F). In KEGG Level 2 (Figure 3D), the five most prominent marker pathways showed clear regional differentiation. The YZ and SG groups were predominantly enriched in energy metabolism-related pathways, while the KT group showed distinct enrichment in membrane transport and cellular processes. In contrast, the TLXG group exhibited higher enrichment in pathways related to genetic information processing, such as major categories including replication and repair, translation, etc. In CAZy (Figure 3C), region-specific enrichment patterns were likewise observed among GH, GT and CE families, indicating differentiated potential in carbohydrate degradation capabilities among geographic populations. Based on CARD annotations (Figure 3E), populations displayed distinguishable compositions of database-inferred resistance-related gene markers at the ARO and antibiotic-class levels. The WY group was relatively enriched for multidrug and aminoglycoside-related markers, and SG group showed higher levels in entries related to peptide- and nucleoside-related categories, whereas TheYZ and GZ09_11 groups presented a more diverse and broad-spectrum combination of resistance-related markers. In VFDB (Figure 3F), the populations also differed in the composition of homology-based virulence-factor categories, suggesting variation in virulence-associated genetic potential.

In summary, populations from different ecological regions exhibited pronounced functional differentiation across multiple layers—core metabolic functions, CAZy family composition, and health-associated reference sets (antibiotic- and virulence-related markers). Taken together, these findings indicate regionally shaped functional configurations of the Przewalski’s gazelle gut microbiome under host- and environment-specific selective pressures, highlighting tight links between microbial function and ecological adaptation in this endangered species.

### 3.4. Correlation Analysis Between Gut Microbiota Composition and Functional Profiles in Przewalski’s Gazelles Across Different Regions

**Figure 4:**
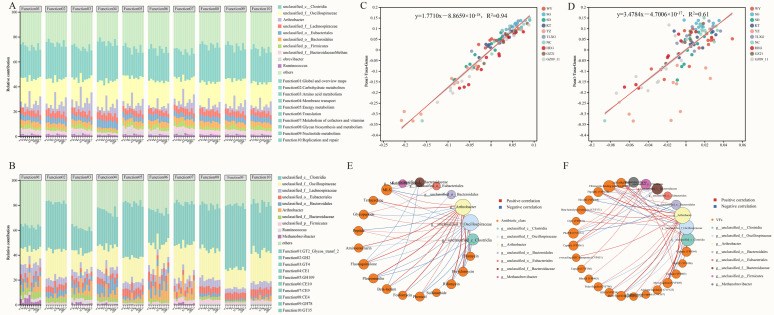
Correlation analyses between gut microbiota composition and functions across regions in Przewalski’s gazelle. (**A**) Taxon contributions to KEGG Level 2 functions; (**B**) Taxon contributions to CAZy family functions; (**C**) Linear regression between genus-level β-diversity and KEGG Level 2 functional β-diversity; (**D**) Linear regression between genus-level β-diversity and CAZy family functional β-diversity; (**E**) Species–antibiotic class correlation network (CARD); (**F**) Species–virulence-factor correlation network (VFDB).

To further elucidate the driving mechanisms underlying the functional composition of the gut microbiota in Przewalski’s gazelle, this study evaluated the relative contributions of bacterial genera to functional expression. The results showed that at the KEGG Level 2 pathway level (Figure 4A). The major contributors included taxa such as unclassified Bacteroidaceae/Bacteroidales, unclassified Oscillospiraceae/Lachnospiraceae, *Ruminococcus*, *Methanobrevibacter*, and *Arthrobacter*, which together supported key Level 2 functions including carbohydrate metabolism, amino acid metabolism, membrane transport, and global and overview maps. At the CAZy Family level (Figure 4B), the functional expression of carbohydrate-active enzyme families, such as GT2, GH2, GH13, CE1, and GH78—were predominantly attributed to the same core taxa, highlighting their central ecological role in the degradation and utilization of complex carbon sources.

To further validate the correspondence between the gut microbiota composition and functional profiles of Przewalski’s gazelle, this study performed regression analyses to assess the correlation between microbial composition at the genus level and KEGG Level 2 functions (Figure 4C) as well as CAZy Family functions (Figure 4D). Both relationships were significant, with coefficients of determination R^2^ = 0.61 (KEGG Level 2) and R^2^ = 0.94 (CAZy family), respectively. The tighter fit for CAZy indicates a stronger explanatory and driving effect of community structure in predicting carbohydrate-active enzyme functions, suggesting that the gut microbiota plays a critical role in the formation and regulation of carbon-source metabolic functions.

Based on the correlation network between species and antibiotic classes and between species and virulence factors, we observed a concentrated set of high-magnitude associations that structure the co-variation landscape (Table S3). *Arthrobacter* emerged as a central hub, correlating positively with multiple antibiotic classes—Bicyclomycin (*r* = 0.947), Rifamycin (*r* = 0.905), and Fosfomycin (*r* = 0.849)—and with key virulence factors—MymA operon (*r* = 0.947), FbpABC (*r* = 0.871), and polar flagella (*r* = 0.852)—while showing negative links to Glycopeptide (*r* = −0.664), LOS (*r* = −0.763), and Capsule (*r* = −0.758) (Figure 4E). Bacteroidales/Bacteroidaceae displayed notable negative correlations with MLS (*r* = −0.753). In contrast, unclassified Oscillospiraceae showed strong positive associations with LOS (*r* = 0.821), Capsule (*r* = 0.805), and alginate regulation (*r* = 0.753), together with a strong negative correlation with FbpABC (*r* = −0.742). Taken together, these patterns indicate that a small set of core taxa—particularly *Arthrobacter* and Oscillospiraceae/Bacteroidales lineages—organizes the dominant covariation structure across resistance- and virulence-related categories (Figure 4F).

## 4. Discussion

### 4.1. Metabolic Functional Composition of Gut Microbiota in Different Geographical Populations of Przewalski’s Gazelle

Currently, metagenomics and high-throughput sequencing technologies are widely applied to investigate the structure and function of gut microbiota in ruminants, as well as to assess their impacts on host nutrition and health [16,17]. In this study, we applied metagenomic sequencing to compare functional composition, metabolic pathways, and antibiotic-resistance gene repertoires of Przewalski’s gazelles across regions.

This study found that the gut microbiota of Przewalski’s gazelles from different geographic regions was consistently enriched in core metabolic pathways, particularly in functional modules related to amino acid metabolism, carbohydrate metabolism, and the glycan biosynthesis and metabolism. This suggests a high degree of functional conservation in energy acquisition and nutrient transformation within the gut microbial communities of this species. Previous studies have demonstrated that the gut microbiota of herbivores contributes to host nutrient absorption and ecological adaptation through amino acid metabolism, and degrades plant cell wall polysaccharides via CAZymes (e.g., cellulases, hemicellulases). These functions are considered highly conserved across herbivorous species and geographic regions [18,19]. Consistent with these findings, the results of this study further indicate that the functional stability of core metabolic processes may represent a key gut microbial adaptation mechanism enabling the host to cope with limited nutritional resources and high energy demands in harsh environments.

In the module of genetic information processing, the prominence of functions such as translation and DNA replication/repair reflects the gut microbiota’s strong capacity for community renewal and environmental responsiveness. These features contribute to maintaining intestinal homeostasis in the host and enhancing its resilience to extreme environmental stressors [20,21]. Furthermore, functional annotation based on the CAZy database revealed both geographic variation and functional consistency in the composition of carbohydrate-active enzymes (CAZymes) within the gut microbiota of Przewalski’s gazelle. The analysis showed that across different geographic regions, the dominant CAZyme classes were glycoside hydrolases (GHs) and glycosyl transferases (GTs), with GHs accounting for the highest relative abundance—exceeding 50%, which indicates a broadly conserved capacity among Przewalski’s gazelle gut microbes for degrading complex carbohydrate substrates. Previous studies have reported similar patterns. For instance, based on 16S rRNA gene sequencing and metagenomic analysis, it was found that the abundance of GHs-related genes in the gut microbiota of Przewalski’s horses (Equus przewalskii) was significantly higher in individuals reintroduced into open environments (e.g., Xinjiang Kalamaili Nature Reserve) compared to those in closed habitats (e.g., Anxi Extreme-Arid Desert Nature Reserve), suggesting that carbohydrate metabolism functions are closely linked to habitat conditions [22]. Additionally, metagenomic studies have shown that the gut microbiota of the giant panda harbors GH genes, such as cellulases and β-glucosidases derived from the genus Clostridium, which, although present in low abundance, may compensate for the host’s lack of endogenous cellulose-degrading enzymes and partially enable the digestion of bamboo fibers [23]. Together, these findings and the present study highlight that glycoside hydrolases play essential roles in carbohydrate metabolism within the gut microbiota of wild animals across diverse diets and habitats. Their abundance and diversity not only reflect the host’s adaptive capacity to plant-based diets but are also shaped by environmental pressures and the host’s evolutionary history.

### 4.2. Antibiotic Resistance and Virulence Factor-Related Functions of Gut Microbiota in Different Geographical Populations of Przewalski’s Gazelle

In terms of antibiotic resistance, this study used the CARD database to annotate the Antibiotic Resistance Ontology (ARO) of the gut microbiota of Przewalski’s gazelle (*Procapra przewalskii*), systematically revealing regional differences and indicative genetic signals in the composition of resistance- and virulence-associated genotypic markers. The results showed that Przewalski’s gazelle populations from different regions shared a large set of core antibiotic-related reference markers, with 857 ARO entries detected across all samples, accounting for 77.56% of the total abundance. This indicates that their gut microbiota exhibits a stable and widespread genetic potential associated with antibiotic resistance. Previous studies have shown that, based on metagenomic analysis and CARD annotation, the abundance and diversity of antibiotic resistance genes (ARGs) in the gut microbiota of the giant panda vary significantly depending on the bamboo parts consumed [24]. Specifically, ARG abundance was highest during bamboo leaf consumption, while ARG diversity peaked during bamboo shoot consumption, suggesting that resistance risks may be linked to foodborne environmental contamination [24]. Similarly, metagenomic sequencing and CARD-based analysis of rabbit gut microbiota revealed the presence of multiple antibiotic resistance genes (such as β-lactamases and plasmid-mediated quinolone resistance proteins). Notably, probiotic-treated groups exhibited higher numbers of ARO types, suggesting that probiotics may serve as a potential alternative to antibiotics by promoting growth and modulating resistance gene expression [25]. Collectively, these findings, together with the current study, indicate that both wild and domesticated animals commonly harbor diverse ARGs in their gut microbiota. The composition and abundance of these genes are influenced by geographical conditions, dietary sources, and anthropogenic factors, underscoring the importance of environmental exposure and microbial ecological regulation in the dissemination and modulation of antibiotic resistance genes.

In addition, the analysis of antibiotic class-level distribution in this study revealed that resistance genes related to multidrug resistance (Multidrug), macrolide-lincosamide-streptogramin (MLS), tetracyclines, glycopeptides, and peptides were predominant across all geographical populations of Przewalski’s gazelle. This finding suggests that these may represent commonly occurring antibiotic resistance–related genotypic marker modules within the gut microbiota of Przewalski’s gazelle. Their detection indicates a potential risk of dissemination and zoonotic transmission, which warrants particular attention in regions where Przewalski’s gazelle coexists with grazing livestock. Previous research using metagenomic sequencing and genome-resolved analysis found that multidrug resistance (MDR)-associated genes are widespread in the gut microbiota of wild giant pandas, and their abundance was closely linked to environmental metal pollution, indicating potential resistance risks driven by environmental exposure [26]. Similarly, 16S rRNA and metagenomic studies showed that Himalayan langurs (*Semnopithecus schistaceus*) inhabiting mid-elevation areas harbored a higher abundance of multidrug resistance genes in their gut microbiota, while Xizang macaques (*Macaca mulatta vestita*) from higher elevations exhibited a broader resistance profile, reflecting microbial adaptation to differing environmental pressures. Moreover, tetracycline resistance genes have been widely documented in the gut microbiota of vertebrates, particularly in captive individuals, where their elevated abundance is associated with potential horizontal gene transfer in human-shared environments—posing a dual challenge to public health and species conservation [27]. A metagenomic study of captive primates in Guangxi, China, found that tetracycline resistance genes were more abundant than in wild counterparts, suggesting that lifestyle and habitat conditions significantly shape resistance characteristics and may exacerbate the risk of ARG dissemination [28]. Additionally, metagenomic analyses and CARD-based annotations identified widespread presence of tetracycline and glycopeptide resistance genes in the gut microbiota of relict gulls (*Larus relictus*) and Anatidae species, with the former also harboring a higher number of pathogenic bacteria—indicating elevated ecological risks related to resistance transmission and population health [29]. Together, these findings, consistent with the present study, demonstrate that tetracycline, glycopeptide, and multidrug resistance genes are widely detected across the gut microbiomes of various wild animal species. Their composition and abundance are likely influenced by multiple factors, including environmental contamination, cohabitation with grazing livestock, and habitat conditions.

This study’s analysis of virulence factor (VF) composition revealed that lipooligosaccharides (LOS), fibronectin-binding proteins (FnBP), and the FbpABC transport system consistently maintained high relative abundance across all Przewalski’s gazelle populations, forming a potential core framework of virulence-related reference markers. These virulence factors have been well-documented in both human and animal pathogens for their critical roles in adhesion, invasion, and host colonization processes [30]. Their widespread presence in Przewalski’s gazelle suggests that the gut microbiota of this species may carry a certain degree of virulence-related genetic potential, which may pose health risks under conditions of host stress, immune suppression, or environmental disturbance. Previous studies have shown that captive environments significantly increase the diversity of virulence factors within the gut microbiota of wolves (*Canis lupus*), potentially enhancing their microbial pathogenic potential. This finding underscores the importance of strengthening health monitoring and management in captive individuals [31]. Overall, this study not only revealed that the gut microbiota of Przewalski’s gazelle exhibits both inter-population heterogeneity and a relatively stable core composition in terms of antibiotic resistance– and virulence-related reference genetic markers, but also provides essential baseline information for evaluating wildlife microecological safety, potential zoonotic risks, and the dissemination potential of resistance genes. Future studies should integrate functional validation approaches (e.g., transcriptional or phenotypic assays) with environmental exposure data to elucidate the origins, expression patterns, and dissemination mechanisms of resistance- and virulence-related genes in greater depth.

### 4.3. Functional Differences in Gut Microbiota Among Geographically Distinct Populations of Przewalski’s Gazelle

The analysis of β-diversity in this study revealed significant functional differences in the gut microbiota of Przewalski’s gazelle across multiple functional dimensions (KEGG, CAZy, CARD, VFDB) among different geographic populations. This indicates that metabolic potential, ARG category composition, and virulence-factor marker profiles have undergone regional differentiation.. Previous studies based on 16S rRNA sequencing revealed significant variation in gut microbiota among geographically distinct Przewalski’s gazelle populations, likely driven by differences in local vegetation that shape dietary composition and consequently influence microbial community structure and functional adaptation [8]. Compared with the closely related Tibetan gazelle (*P. picticaudata*), analyses of gut microbiota composition and function indicate that Przewalski’s gazelle exhibits greater variability among geographic populations in dominant taxa abundance, α- and β-diversity, and metabolic functional composition, with significantly lower shared OTU proportions between populations. Neutral community modeling further suggests weak spatial interaction and population connectivity among these small populations [9]. Additionally, studies using DNA barcoding, 16S rRNA sequencing, and metagenomics revealed that seasonal and regional nutritional changes markedly influence the gut microbiota composition and function of wild ungulates such as sika deer (*Cervus nippon*), indicating that gut microbes adjust metabolic pathways to cope with spatiotemporal variations in diet and environment, thereby aiding host nutrition acquisition and environmental adaptation [32]. Non-invasive sampling combined with 16S rRNA sequencing and functional prediction also identified significant differences in gut microbiota composition and metabolic functions among Bengal tiger (*Panthera tigris tigris*) populations from different geographic regions in Nepal, which correlated with host genetic structure, suggesting a key role of gut microbiota in metabolic regulation and environmental adaptation, potentially co-evolving with host gene flow [33]. Taken together, these findings suggest that dietary differences arising from region-specific food resource availability are key drivers of the pronounced compositional and functional differentiation of gut microbiota among Przewalski’s gazelle populations. Such variation not only reflects regional divergence in microbial metabolic potential, resistance mechanisms, and virulence-factor marker profiles, but is also closely linked to limited population connectivity and local environmental adaptation. This further supports the notion that gut microbiota acts as an essential mediator of host environmental adaptation and evolutionary processes, carrying broad ecological significance for endangered ungulates and other wildlife species.

This study systematically compared the functional composition of gut microbiota across different geographic populations of Przewalski’s gazelle and identified significant regional differences at multiple functional levels, including metabolic pathways, carbohydrate-active enzymes, antibiotic resistance genes, and virulence factors. KEGG Level 2 functional analysis revealed highly significant interpopulation differences in core metabolic modules such as Carbohydrate metabolism and Amino acid metabolism, suggesting that metabolic potential may have undergone divergent adaptation under varying environmental conditions. Differences at the CAZy family level further indicated functional differentiation in carbohydrate degradation and utilization strategies among populations, reflecting microbial regional adaptation in energy acquisition mechanisms. Previous research has demonstrated, through metabolomic screening combined with gene function validation, that multiple amino acid metabolism-related genes in the gut microbiota significantly regulate intestinal and circulating amino acid levels, thereby influencing host nutritional homeostasis and physiological functions [34]. Metagenomic sequencing analyses have shown that gut microbiota of Francois’ langurs (*Trachypithecus francoisi*) exhibit significant variation in carbohydrate and amino acid metabolism-related functional genes under different environmental and dietary conditions, with carbohydrate metabolism genes notably enriched in individuals exposed to anthropogenic disturbance and captivity, indicating potential microbial plasticity in regulating metabolism to adapt to diverse diets [35]. Collectively, integrating the results of this study with existing literature suggests that gut microbiota of Przewalski’s gazelle from different geographic populations displays significant regional functional differentiation in core metabolic functions particularly carbohydrate and amino acid metabolism—reflecting high ecological adaptability and environmental plasticity through metabolic regulatory mechanisms, thereby facilitating host adaptation to diverse ecological environments.

Additionally, based on LEfSe analysis, this study found significant differences among populations in the composition of antibiotic resistance genes and virulence factors, with certain groups such as WY and SG groups exhibiting enrichment in specific resistance categories. This may reflect antibiotic exposure pressures in their environments or differences in host life history traits. Previous studies have reported that metagenomic sequencing of gut microbiomes from captive and rescued pangolins revealed a significant enrichment of virulence factors in captive individuals, suggesting that captivity may increase potential pathogenic risks and necessitate enhanced health management and disease prevention [36]. Through 16S rRNA gene sequencing and metagenomic analysis, it was found that virulence factors were significantly enriched in the gut microbiota of captive giant pandas, suggesting that the captive environment may enhance genotypic marker signals associated with pathogenicity and potentially affect post-release adaptability and ecosystem health [37]. Integrating these findings, the pronounced differences in resistance genes and virulence factors among gut microbiota of different Przewalski’s gazelle populations likely reflect the influence of environmental antibiotic exposure and host life history. In summary, the gut microbiota of Przewalski’s gazelle across distinct geographic populations exhibits a complex and region-specific functional differentiation pattern, suggesting its critical role in mediating host responses to environmental heterogeneity, regulating metabolic adaptability, and resisting external stressors.

### 4.4. Correlation Between Gut Microbial Composition and Functional Profiles of Przewalski’s Gazelle Across Different Regions

This study systematically revealed a strong coupling between the genus-level composition and functional profiles of the gut microbiota in Przewalski’s gazelle, highlighting the pivotal roles of core taxa such as unclassified Bacteroidaceae/Bacteroidales, unclassified Oscillospiraceae/Lachnospiraceae, *Ruminococcus*, *Methanobrevibacter*, and *Arthrobacter* in core metabolic functions—including carbohydrate metabolism, amino acid metabolism, and membrane transport—as well as in CAZy-associated glycosyltransferase and glycoside hydrolase activities. These findings further confirm the critical influence of microbial community structure on the degradation of complex carbon sources and energy metabolism functions. Regression analyses demonstrated a significant positive correlation between microbial composition and functional spectra, with stronger explanatory power observed in carbohydrate-metabolizing enzyme activities, underscoring the decisive role of gut microbiota structure in functional regulation. Previous studies have indicated that gut microbial composition is closely linked to host metabolic functions, where diverse dietary nutrients modulate microbial communities and significantly impact microbial metabolic activity and host health. This emphasizes the importance of precise quantification and analysis of diet-related gut microbiota variables to elucidate the interactions between microbiota and metabolic functions [38]. Using 16S rRNA gene sequencing combined with partial least squares discriminant analysis (PLS-DA), it was shown that diet exerts a much greater effect on murine gut microbiota structure than genetics, and significant shifts in gut microbiota—especially the depletion of protective Bifidobacterium and enrichment of endotoxin-producing bacteria—were closely associated with metabolic syndrome, highlighting the critical role of gut microbiota in regulating host metabolism [39]. Dietary intervention experiments revealed that although overall gut microbiota composition remained stable, cellulose-degrading and nitrogen-fixing microbial taxa were significantly enriched in metabolically elevated viviparous cockroaches (*Diploptera punctata*), suggesting that gut microbial metabolic functions, particularly essential amino acid provision, closely correlate with host basal metabolic rate [40]. Multi-omics integration of 16S rRNA sequencing with host tissue RNA-Seq demonstrated that specific gut microbes are closely associated with dysregulated expression of inflammation-related cytokines (e.g., IL-17, IL-23, IFNγ), and these microbes modulate host immunity by disturbing metabolic pathways such as short-chain fatty acid and lipopolysaccharide synthesis, underscoring the tight link between gut microbial metabolism and immune regulation [41]. Functional prediction analyses of 16S rRNA data showed that captive golden snub-nosed monkeys (*Rhinopithecus roxellana*) exhibit gut microbiota structures significantly distinct from wild individuals, with carbohydrate metabolism pathways exhibiting the most pronounced differences, indicating diet-driven gut microbiota shifts closely tied to metabolic function regulation [42]. Collectively, this study, together with existing literature, demonstrates that the gut microbiota structure and metabolic functions of Przewalski’s gazelle are highly coupled, with dominant genera acting as key drivers of core metabolic pathways and carbohydrate-active enzyme functions. Changes in gut microbial composition not only profoundly influence host metabolic homeostasis but also participate in immune modulation through the regulation of metabolic products and immune factors, highlighting the central role of gut microbiota in host energy metabolism and immune balance. In future research, identifying Przewalski’s gazelle populations with elevated resistome or virulence burdens in areas overlapping with pastoral activities could support targeted microbial health monitoring and guide co-grazing management strategies. Moreover, integrating regional dietary data with CAZy enzyme profiles will help elucidate how forage composition and feeding habits drive microbial carbohydrate metabolism and functional differentiation across populations.

This study, integrating annotations of antibiotic resistance genes and virulence factors from the CARD and VFDB databases, revealed significant associations between core bacterial genera and multiple antibiotic resistance mechanisms as well as virulence-related modules. These findings indicate that the structural dynamics of the gut microbiota play an important regulatory role in shaping the genotypic marker profiles associated with resistance and virulence. Previous research analyzing metagenomic sequencing and resistance genes from 122 cecal samples across five rodent species demonstrated a significant correlation between gut microbiota composition and the antibiotic resistance gene (ARG) profile [43]. Multidrug resistance genes were primarily carried by core taxa such as Oscillospiraceae, highlighting the key role of specific gut microbial groups in ARG dissemination [43]. Using UPLC-MS metabolomics, 16S rRNA sequencing, and metagenomic analyses, studies on giant pandas found that with increasing age and dietary shifts, gut microbiota structure and function changed significantly, with the number and diversity of ARGs progressively increasing [44]. Certain potential pathogens were enriched during juvenile stages, revealing an age-dependent role of the gut microbiota in antibiotic resistance spread and pathogenic potential development [44]. Through combined 16S rRNA and whole genome sequencing (WGS) analyses, endangered Galapagos penguins (*Spheniscus mendiculus*) were found to harbor widespread ARGs and virulence factors (VFs) within their gut microbiota [45]. The diversity of microbial structure and function was notably influenced by individual developmental stages and the presence of potential pathogen Clostridium perfringens, underscoring the important ecological role of gut microbiota in resistance gene dissemination and pathogenic potential formation [45]. By integrating the findings of this study with previous research, it can be inferred that the dynamic coupling between gut microbial community structure and functional annotation patterns is closely linked to the genotypic marker profiles associated with resistance and virulence. Variations across species, developmental stages, and core bacterial genera can markedly modulate the antibiotic resistance level and genetic potential landscape of the gut microbiota, highlighting its critical ecological role in shaping host health and disease risk. Overall, this study deepens the understanding of the co-evolutionary mechanisms between gut microbiota composition and function in Przewalski’s gazelle, demonstrating that microbial community structure not only determines metabolic functional expression but also contributes to the formation of resistance- and virulence-associated health risks. This provides novel perspectives and theoretical support for ecological adaptation and conservation biology. Future research should further integrate host phenotypic traits and environmental variables to elucidate the specific mechanisms by which these microecological functional traits influence population health and environmental adaptation.The findings and their implications should be discussed in the broadest context possible. Future research directions may also be highlighted.

## 5. Conclusions

This study employed metagenomics to systematically reveal a functional pattern in the gut microbiota of distinct geographical populations of Przewalski’s gazelle, characterized by a conserved core with population-specific divergence. Although the gut microbial communities were highly conserved in core energy-acquisition pathways such as carbohydrate and amino acid metabolism, the composition and distribution of antibiotic- and virulence-related marker profiles exhibited significant regional differentiation, which was closely associated with varying local ecological pressures and anthropogenic disturbances. Furthermore, key bacterial genera (e.g., *Arthrobacter* and Oscillospiraceae/Bacteroidales lineages) were pinpointed as crucial drivers of core metabolic, resistance, and virulence functions, forming a critical bridge linking microbial community structure to host ecological adaptation. Overall, the functional plasticity of the gut microbiota in Przewalski’s gazelle plays a pivotal role in facilitating host adaptation to heterogeneous habitats. These findings not only deepen our understanding of the microecological adaptation mechanisms in wild endangered animals but also provide a vital scientific basis for formulating targeted, population-specific conservation and management strategies. 

## Data Availability

The gut microbiota data of *Procapra przewalskii* generated and analyzed in this study are currently undergoing further in-depth analysis for subsequent research. Therefore, the data are not publicly available at this time but may be made accessible upon reasonable request after the completion of ongoing studies.

## Supplementary Materials

The following supporting information can be downloaded at: https://www.mdpi.com/article/10.3390/microorganisms13112513/s1, Table S1: Summary of sampling sites and associated metadata. Table S2: Kruskal–Wallis statistics and ε2 effect sizes for KEGG Level 2 and CAZy families. Table S3: Genus–function correlation results (CARD antibiotic classes and virulence factors): node pairs, correlation coefficients, and significance.
