# Supplementary material for: Metagenome-Based Functional Differentiation of Gut Microbiota and Ecological Adaptation Among Geographically Distinct Populations of Przewalski’s Gazelle (Procapra przewalskii)
Source: Microorganisms. 2025 Oct 31;13(11):2513. doi: 10.3390/microorganisms13112513 (PMC12654606; doi:10.3390/microorganisms13112513)
Supplement: Supplementary file 1 [file microorganisms-13-02513-s001.zip › microorganisms-3932112-supplementary.pdf]

Table S1. Summary of sampling sites and associated metadata

Group	Name	Latitude	Longitude	Altitude	Population Size	Season
GZ21	GZH4821	37.12430804	100.472266	3235.604	136	Spring
GZ09_11	GZH4809	37.15958102	100.518835	3275.892	136	Spring
KT	KT4770	36.80331702	100.80196	3255.68	12	Spring
WY	WY4850	36.56424302	99.306236	3083.894	77	Spring
YZ	YZ4855	36.58634501	99.370551	3225.238	12	Spring
HEG	HEG4837	37.18225096	100.434319	3249.751	99	Spring
SD	SD4796	36.86915	100.741666	3234.449	134	Spring
NC	NC4829	37.18657804	100.361078	3226.451	1235	Spring
SG	SG4844	37.49670703	98.521079	3701.423	46	Spring
TLXG	TLXG4788	37.18084901	100.566593	3324.134	59	Spring

Table S2. Kruskal–Wallis statistics and ε² effect sizes for KEGG Level 2 and CAZy families

Function	Name	k	df	n	epsilon2-kw	epsilon2-CI95-low	epsilon2-CI95-high	*p* value	Corrected *p* value
CAZY-Family	GT2_Glycos_transf_2	10	9	105	0.2118	0.1222	0.4444	1.12E-03	2.59E-03
CAZY-Family	GH2	10	9	105	0.2415	0.1379	0.4999	3.80E-04	1.03E-03
CAZY-Family	GT4	10	9	105	0.3471	0.2726	0.5241	3.05E-03	6.26E-03
CAZY-Family	CE1	10	9	105	0.4535	0.3452	0.6291	1.60E-05	7.13E-05
CAZY-Family	GH109	10	9	105	0.1330	0.0476	0.3846	2.81E-04	8.06E-04
CAZY-Family	CE10	10	9	105	0.3018	0.2135	0.5002	6.84E-06	3.57E-05
CAZY-Family	CE9	10	9	105	0.1447	0.0797	0.3715	3.57E-06	2.25E-05
CAZY-Family	CE4	10	9	105	0.3711	0.2697	0.5894	9.74E-07	8.86E-06
CAZY-Family	GH78	10	9	105	0.1375	0.0470	0.4011	2.74E-02	4.07E-02
CAZY-Family	GT35	10	9	105	0.4199	0.3253	0.6090	8.08E-05	2.77E-04
KEGG-Level2	Global and overview maps	10	9	105	0.3340	0.2389	0.5384	2.28E-09	3.58E-08
KEGG-Level2	Carbohydrate metabolism	10	9	105	0.2920	0.2232	0.5137	1.87E-05	2.58E-05
KEGG-Level2	Amino acid metabolism	10	9	105	0.3761	0.2725	0.5895	1.13E-08	1.06E-07
KEGG-Level2	Membrane transport	10	9	105	0.1518	0.0858	0.3813	5.81E-05	7.38E-05
KEGG-Level2	Energy metabolism	10	9	105	0.3555	0.2427	0.5784	2.98E-07	9.99E-07
KEGG-Level2	Translation	10	9	105	0.3553	0.2763	0.5452	3.88E-07	1.14E-06

KEGG-Level2	Metabolism of cofactors and vitamins	10	9	105	0.4689	0.3734	0.6560	2.61E-10	1.23E-08
KEGG-Level2	Glycan biosynthesis and metabolism	10	9	105	0.3110	0.2174	0.5419	2.50E-05	3.36E-05
KEGG-Level2	Nucleotide metabolism	10	9	105	0.2461	0.1665	0.4610	5.70E-05	7.38E-05
KEGG-Level2	Replication and repair	10	9	105	0.3806	0.2953	0.5810	3.52E-04	4.13E-04

Table S3. Genus–function correlation results (CARD antibiotic classes and virulence factors): node pairs, correlation coefficients, and significance

Functional Category	Node1_Name	Node2_Name	Coefficient	Pvalue
CARD antibiotic classes	g__unclassified_c__Clostridia|p__Firmicutes	Peptide	0.638	2.59E-13
CARD antibiotic classes	g__unclassified_c__Clostridia|p__Firmicutes	Pleuromutilin	-0.709	2.46E-17
CARD antibiotic classes	g__unclassified_c__Clostridia|p__Firmicutes	Phenicol	-0.732	6.89E-19
CARD antibiotic classes	g__unclassified_c__Clostridia|p__Firmicutes	Sulfonamide	-0.613	3.78E-12
CARD antibiotic classes	g__unclassified_c__Clostridia|p__Firmicutes	Rifamycin	-0.622	1.41E-12
CARD antibiotic classes	g__unclassified_c__Clostridia|p__Firmicutes	Bicyclomycin	-0.697	1.43E-16
CARD antibiotic classes	Peptide	g__unclassified_f__Oscillospiraceae|p__Firmicutes	0.626	9.11E-13
CARD antibiotic classes	Peptide	g__Arthrobacter|p__Actinobacteria	-0.711	2.00E-17
CARD antibiotic classes	Pleuromutilin	g__unclassified_f__Oscillospiraceae|p__Firmicutes	-0.690	4.17E-16
CARD antibiotic classes	Pleuromutilin	g__Arthrobacter|p__Actinobacteria	0.853	7.55E-31
CARD antibiotic classes	Phenicol	g__unclassified_f__Oscillospiraceae|p__Firmicutes	-0.712	1.72E-17
CARD antibiotic classes	Phenicol	g__Arthrobacter|p__Actinobacteria	0.669	5.93E-15
CARD antibiotic classes	Rifamycin	g__unclassified_f__Oscillospiraceae|p__Firmicutes	-0.754	1.60E-20
CARD antibiotic classes	Rifamycin	g__Arthrobacter|p__Actinobacteria	0.905	4.27E-40
CARD antibiotic classes	Bicyclomycin	g__unclassified_f__Oscillospiraceae|p__Firmicutes	-0.778	1.70E-22

CARD antibiotic classes	Bicyclomycin	g__Arthrobacter|p__Actinobacteria	0.947	8.69E-53
CARD antibiotic classes	g__unclassified_f__Oscillospiraceae|p__Firmicutes	MLS	-0.645	1.06E-13
CARD antibiotic classes	g__unclassified_f__Oscillospiraceae|p__Firmicutes	Tetracycline	-0.639	2.19E-13
CARD antibiotic classes	g__unclassified_f__Oscillospiraceae|p__Firmicutes	Glycopeptide	0.647	8.38E-14
CARD antibiotic classes	g__unclassified_f__Oscillospiraceae|p__Firmicutes	Aminocoumarin	-0.601	1.25E-11
CARD antibiotic classes	g__unclassified_f__Oscillospiraceae|p__Firmicutes	Fluoroquinolone	-0.636	2.98E-13
CARD antibiotic classes	g__unclassified_f__Oscillospiraceae|p__Firmicutes	Beta-lactam	-0.681	1.35E-15
CARD antibiotic classes	g__unclassified_f__Oscillospiraceae|p__Firmicutes	Fosfomycin	-0.670	5.60E-15
CARD antibiotic classes	g__unclassified_f__Oscillospiraceae|p__Firmicutes	Elfamycin	-0.664	1.12E-14
CARD antibiotic classes	MLS	g__Arthrobacter|p__Actinobacteria	0.688	4.89E-16
CARD antibiotic classes	MLS	g__unclassified_o__Bacteroidales|p__Bacteroidota	-0.753	2.11E-20
CARD antibiotic classes	MLS	g__unclassified_f__Bacteroidaceae|p__Bacteroidota	-0.699	1.18E-16
CARD antibiotic classes	MLS	g__Methanobrevibacter|p__Euryarchaeota	0.614	3.39E-12
CARD antibiotic classes	Tetracycline	g__Arthrobacter|p__Actinobacteria	0.794	4.97E-24
CARD antibiotic classes	Glycopeptide	g__Arthrobacter|p__Actinobacteria	-0.664	1.12E-14
CARD antibiotic classes	Glycopeptide	g__unclassified_o__Eubacteriales|p__Firmicutes	0.629	6.91E-13

CARD antibiotic classes	Aminocoumarin	g__Arthrobacter|p__Actinobacteria	0.782	7.53E-23
CARD antibiotic classes	Fluoroquinolone	g__Arthrobacter|p__Actinobacteria	0.793	6.54E-24
CARD antibiotic classes	Fluoroquinolone	g__unclassified_o__Bacteroidales|p__Bacteroidota	-0.621	1.62E-12
CARD antibiotic classes	Fluoroquinolone	g__unclassified_f__Bacteroidaceae|p__Bacteroidota	-0.659	2.05E-14
CARD antibiotic classes	Beta-lactam	g__Arthrobacter|p__Actinobacteria	0.741	1.51E-19
CARD antibiotic classes	Beta-lactam	g__unclassified_f__Bacteroidaceae|p__Bacteroidota	-0.689	4.26E-16
CARD antibiotic classes	Fosfomycin	g__Arthrobacter|p__Actinobacteria	0.849	2.96E-30
CARD antibiotic classes	Elfamycin	g__Arthrobacter|p__Actinobacteria	0.823	4.67E-27
VFDB virulence-factor	g__unclassified_c__Clostridia|p__Firmicutes	LOS (CVF494)	0.701	8.45E-17
VFDB virulence-factor	g__unclassified_c__Clostridia|p__Firmicutes	Fibronectin-binding protein (AI238)	0.629	6.51E-13
VFDB virulence-factor	g__unclassified_c__Clostridia|p__Firmicutes	Polar flagella (VF0473)	-0.607	6.83E-12
VFDB virulence-factor	g__unclassified_c__Clostridia|p__Firmicutes	Polysaccharide capsule (CVF567)	0.622	1.37E-12
VFDB virulence-factor	g__unclassified_c__Clostridia|p__Firmicutes	Alginate regulation (CVF523)	0.665	9.80E-15
VFDB virulence-factor	g__unclassified_c__Clostridia|p__Firmicutes	MymA operon (CVF649)	-0.650	6.37E-14
VFDB virulence-factor	g__unclassified_c__Clostridia|p__Firmicutes	MgtBC (VF0106)	0.714	1.17E-17
VFDB virulence-factor	g__unclassified_c__Clostridia|p__Firmicutes	Capsule (VF0144)	0.685	7.81E-16

VFDB virulence-factor	LOS (CVF494)	g__unclassified_f__Oscillospiraceae|p__Firmicutes	0.821	9.11E-27
VFDB virulence-factor	LOS (CVF494)	g__Arthrobacter|p__Actinobacteria	-0.763	3.06E-21
VFDB virulence-factor	Fibronectin-binding protein (AI238)	g__unclassified_f__Oscillospiraceae|p__Firmicutes	0.716	9.63E-18
VFDB virulence-factor	Fibronectin-binding protein (AI238)	g__Arthrobacter|p__Actinobacteria	-0.686	7.20E-16
VFDB virulence-factor	Fibronectin-binding protein (AI238)	g__unclassified_o__Bacteroidales|p__Bacteroidota	0.657	2.58E-14
VFDB virulence-factor	Fibronectin-binding protein (AI238)	g__unclassified_f__Bacteroidaceae|p__Bacteroidota	0.724	2.80E-18
VFDB virulence-factor	Fibronectin-binding protein (AI238)	g__Methanobrevibacter|p__Euryarchaeota	-0.668	7.39E-15
VFDB virulence-factor	Polar flagella (VF0473)	g__unclassified_f__Oscillospiraceae|p__Firmicutes	-0.675	2.87E-15
VFDB virulence-factor	Polar flagella (VF0473)	g__Arthrobacter|p__Actinobacteria	0.852	1.09E-30
VFDB virulence-factor	Alginate regulation (CVF523)	g__unclassified_f__Oscillospiraceae|p__Firmicutes	0.753	1.97E-20
VFDB virulence-factor	MymA operon (CVF649)	g__unclassified_f__Oscillospiraceae|p__Firmicutes	-0.774	3.96E-22
VFDB virulence-factor	MymA operon (CVF649)	g__Arthrobacter|p__Actinobacteria	0.947	9.40E-53
VFDB virulence-factor	Capsule (VF0144)	g__unclassified_f__Oscillospiraceae|p__Firmicutes	0.805	4.40E-25
VFDB virulence-factor	Capsule (VF0144)	g__Arthrobacter|p__Actinobacteria	-0.758	8.60E-21
VFDB virulence-factor	g__unclassified_f__Oscillospiraceae|p__Firmicutes	FbpABC (VF0272)	-0.742	1.29E-19
VFDB virulence-factor	g__unclassified_f__Oscillospiraceae|p__Firmicutes	Beta-hemolysin/cytolysin (CVF171)	-0.664	1.14E-14

VFDB virulence-factor	g__unclassified_f__Oscillospiraceae|p__Firmicutes	Polar flagella (CVF786)	-0.638	2.55E-13
VFDB virulence-factor	g__unclassified_f__Oscillospiraceae|p__Firmicutes	HSI-I (VF0334)	-0.608	6.13E-12
VFDB virulence-factor	FbpABC (VF0272)	g__unclassified_f__Bacteroidaceae|p__Bacteroidota	-0.647	8.92E-14
VFDB virulence-factor	FbpABC (VF0272)	g__Arthrobacter|p__Actinobacteria	0.871	1.26E-33
VFDB virulence-factor	Beta-hemolysin/cytolysin (CVF171)	g__Arthrobacter|p__Actinobacteria	0.719	5.62E-18
VFDB virulence-factor	Polar flagella (CVF786)	g__Arthrobacter|p__Actinobacteria	0.649	6.86E-14
VFDB virulence-factor	HSI-I (VF0334)	g__Arthrobacter|p__Actinobacteria	0.724	2.79E-18
VFDB virulence-factor	g__Arthrobacter|p__Actinobacteria	ClpC (VF0072)	0.707	3.26E-17
VFDB virulence-factor	g__Arthrobacter|p__Actinobacteria	Trehalose-recycling ABC transporter (CVF651)	0.716	9.60E-18
VFDB virulence-factor	g__Arthrobacter|p__Actinobacteria	Hsp60 (VF0159)	0.695	2.03E-16
VFDB virulence-factor	g__unclassified_o__Bacteroidales|p__Bacteroidota	HitABC (VF0268)	-0.726	1.90E-18
VFDB virulence-factor	g__unclassified_o__Bacteroidales|p__Bacteroidota	PhoP/R (CVF331)	-0.763	2.97E-21
VFDB virulence-factor	HitABC (VF0268)	g__unclassified_f__Bacteroidaceae|p__Bacteroidota	-0.615	2.99E-12
VFDB virulence-factor	PhoP/R (CVF331)	g__unclassified_f__Bacteroidaceae|p__Bacteroidota	-0.661	1.71E-14
VFDB virulence-factor	PhoP/R (CVF331)	g__unclassified_p__Firmicutes|p__Firmicutes	0.614	3.22E-12
VFDB virulence-factor	PhoP/R (CVF331)	g__Methanobrevibacter|p__Euryarchaeota	0.613	3.52E-12

VFDB virulence-factor	g__unclassified_o__Eubacteriales|p__Firmicutes	BfmRS (VF0463)	0.640	1.93E-13
VFDB virulence-factor	g__unclassified_f__Bacteroidaceae|p__Bacteroidota	Capsule (VF0361)	-0.630	6.07E-13
VFDB virulence-factor	Capsule (VF0361)	g__unclassified_p__Firmicutes|p__Firmicutes	0.707	3.76E-17
VFDB virulence-factor	Capsule (VF0361)	g__Methanobrevibacter|p__Euryarchaeota	0.758	7.47E-21
VFDB virulence-factor	g__unclassified_p__Firmicutes|p__Firmicutes	Capsule (CVF186)	0.669	6.39E-15
VFDB virulence-factor	g__unclassified_p__Firmicutes|p__Firmicutes	Capsule (VF0003)	0.621	1.52E-12
VFDB virulence-factor	Capsule (VF0003)	g__Methanobrevibacter|p__Euryarchaeota	0.689	4.63E-16
